# Impact of the First Wave of COVID-19 Pandemic on Radiotherapy Practice at Tata Memorial Centre, Mumbai: A Longitudinal Cohort Study

**DOI:** 10.1200/GO.21.00365

**Published:** 2022-07-08

**Authors:** Anil Tibdewal, Rima Pathak, Anuj Kumar, Sachith Anand, Sarbani Ghosh Laskar, Rajiv Sarin, Supriya Chopra, Reena Engineer, Siddharth Laskar, Vedang Murthy, Tejpal Gupta, Jai Prakash Agarwal

**Affiliations:** ^1^ Department of Radiation Oncology, Tata Memorial Centre, Homi Bhabha National Institute, Mumbai, India

## Abstract

**PURPOSE:**

Delivery of cancer care during the pandemic required adopting various changes in the standard management. We analyzed the impact of the first wave of the COVID-19 pandemic on radiation oncology treatment practices at Tata Memorial Hospital in India.

**MATERIALS AND METHODS:**

From March 1 to October 31, 2020, all consecutive patients who attended the radiation oncology department for radiotherapy treatment were included in this study. Electronic medical records, patient files, and telephonic consult were used to collect patient's data including changes in the standard treatment practice, COVID-19 testing and its results, and subsequent impact on radiotherapy treatment. Comparison was done with the same period data of 2019 for the number of the caseload, radiotherapy regimen, referral rates, and noncompliance rates.

**RESULTS:**

Our study included 4,256 patients with a median age of 52 years (interquartile range 41-61 years). There was a significant drop in the new-patient registrations (approximately 63%), radiotherapy consultations (44.9%), and referrals to other centers (27.8%). The reduction in the caseload was highest for genitourinary cases (–58.5%) and the lowest for breast cases (–11.5%) when compared with the 2019 cohort. Among those treated with radical intent, the noncompliance rate was 15%. Hypofractionation was the commonly adopted regimen across all sites. Compared with 2019, the maximum reduction in the average fractions per patient was seen in the breast cancer cases (–8.2 fraction), followed by genitourinary cases (–4.9 fraction). Of the 27.8% of patients tested for COVID-19, 13.4% turned positive and 3.4% died due to the disease.

**CONCLUSION:**

The COVID-19 pandemic adversely affected the number of radiotherapy consultations and treatments at our institute. However, our department offered uninterrupted services despite grave challenges. Hypofractionated regimen was used across disease sites to minimize patient visits and allow planned treatment completion. Radiotherapy was delivered safely, and patients experienced low rates of COVID positivity during radiotherapy and even lower mortality.

**CONTEXT**

**Key Objective**
To assess the impact of the first wave of the pandemic on radiation oncology (RO) treatment practices at a tertiary cancer center in India.
**Knowledge Generated**
This study analyses the change in treatment practices at the RO department of the largest tertiary cancer care center in India and the importance of adoption of hypofractionation regimens to minimize treatment visits, thereby improving the compliance and completion of planned treatments.
**Relevance**
The first pandemic wave derailed the health care systems of the country with surge in COVID-19 cases and nationwide lockdowns. However, despite grave challenges, cancer care services were continued without any interruptions at the RO department of a high-volume center with regimens to complete planned treatments earlier than that of standard practice.

## BACKGROUND

The ongoing COVID-19 pandemic has impelled an unprecedented global crisis. The consequences for humanity are grave and far-reaching. COVID-19 cases have crossed 219 million with 4.5 million deaths since the beginning of the outbreak.^1^ The total number of cases in India has crossed 33 million, with over 6.6 lakh casualties.^2,3^ The first pandemic wave in 2020 witnessed a complete lockdown in many countries, with the health care systems in disarray.^4^ In India, sequential lockdowns were introduced from March 25, 2020, to May 31, 2020, when all transportation facilities were aborted, except for the delivery of essential services. During this period, cancer care services were also drastically affected.^5^ Many hospitals, including reputed cancer centers, were partially or entirely transformed into COVID hospitals, and the staff were forced to provide COVID care.^6^

During the pandemic, at the Tata Memorial Center, Mumbai, a core COVID-19 action group was created for daily briefings and formulation of action plans. This was deemed necessary because of the meager understanding and the ever-changing COVID scenario in the country requiring adaptation almost daily. Screening camps were established outside our cancer hospital to monitor the temperature and symptoms among patients and caregivers, thereby restricting the potential of infection. Additionally, fever clinics and isolation wards were also set up to treat symptomatic COVID-positive staff and patients with cancer managed at our institute.^7^

Delivery of cancer care, though critical, was not prioritized during this phase of the pandemic across many centers that otherwise actively engage in providing cancer care. Our center is India's largest comprehensive cancer center that registers around 40,000-45,000 new patients with cancer every year. The total number of registrations decreased because of the pandemic and lockdowns. However, our center continued to provide care to patients with cancer in the newly converted COVID isolation wards even when they tested COVID-positive.^8,9^

Our radiation oncology (RO) department is one of the largest in the country with a workforce of nearly 300 professionals, including radiation oncologists, medical physicists, technologists, nurses, administrators, and allied workers. Owing to the protracted nature of radiotherapy (RT), patients, caregivers, and health care providers have a greater probability of contracting infection and experiencing disruptions in treatments if they tested COVID-positive while on RT. Multiple reports indicated that government travel restrictions, lockdowns, and looming fear of infection forced many patients to discontinue treatment.^10^ To limit the patient and caregiver visits to the hospital, thereby reducing their potential exposure and to ease the pressure on the already sparse and fatigued workforce, many national and international guidelines recommended changes in the standard practice.^11-14^

Many modifications were made to the standard prepandemic management practices across most disease sites at various time points. However, the cumulative impact these changes had on the management at a macroscopic level across the department was not known. Our center being among the largest in the country provided a unique opportunity to evaluate the impact of COVID-19 pandemic on RT practice in greater detail.

## MATERIALS AND METHODS

This ambispective observational study was conducted at the RT department of Tata Memorial Hospital, Mumbai, during the first wave of the pandemic which witnessed strict travel restrictions and lockdowns from March 1 to October 31, 2020. All patients who visited the RT department for treatment consultation and active treatment were eligible for participating in the study, whereas those visiting for routine follow-up were excluded. For comparison, we collected anonymized data of the RT courses delivered and patients referred to other centers for RT during the same period (March-October) for the year 2019 and grouped them by their site of primary and therapy intent. These data were obtained from the indigenous reporting and archiving software radiation oncology information system where this information is prospectively entered.

After the institutional ethics approval (Project No: 3548/IEC-I/08-2020), a written informed consent was taken from all prospective patients, and consent waiver was obtained for the retrospective patients. Patients were grouped on the basis of the primary disease site(s) such as head, neck, breast, thorax, gynecology, gastrointestinal, genitourinary, and central nervous system, and pediatric tumors were clubbed with hematolymphoid and bone and soft-tissue tumors as pediatric, hematolymphoid, and bone soft tissue.

Individual patient data points such as demographics, tumor, and treatment details were collected from electronic medical records, files, and telephonic communication and collated in a common datasheet. Any change in the standard investigations and RT practice were recorded. Patients who underwent COVID-19 testing during RT and its results were also noted. We also noted any delay in completion or change in treatment intent among those who turned COVID-19–positive. Patients who wished to receive further treatment at centers closer to their home because of travel restrictions were provided referral letters with treatment summaries for continuity of care as per institutional practice. All the comparisons reported in this article on the treatment practice and throughput between the year 2019 and 2020 pertain to the study period from March 1 to October 31.

All demographic and treatment-related variables were analyzed using Statistical Package for Social Sciences, version 25 (SPSS Inc, Chicago, IL).

## RESULTS

### Impact on Patient Numbers

**FIG 1:**
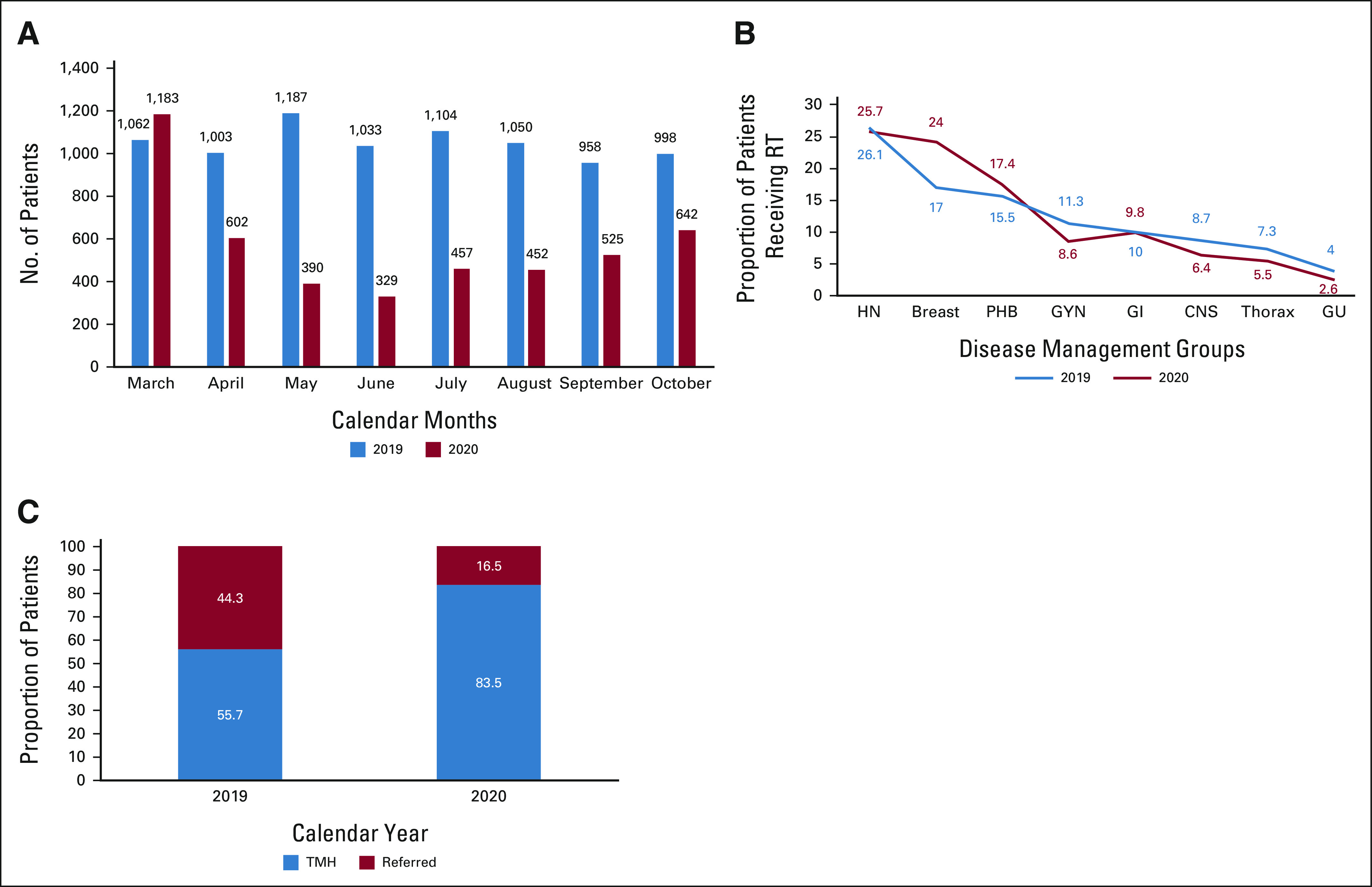
The reduction in (A) monthly consultations and (B) site-wise reduction in patients treated with RT in our department and (C) proportion of patients referred to other centers in 2020 compared with 2019. GU, genitourinary; GYN, gynecological; HN, head and neck; PHB, pediatric, hematolymphoid, and bone soft tissue; RT, radiotherapy.

Approximately 11,000 new patients with cancer registered at our center from March 1 to October 31, 2020. This was significantly (63%) lower compared with 2019, when approximately 30,000 new patients with cancer registered during the same period. A total of 4,256 of 4,580 (93%) patients who visited the RT department for treatment consultation during the study period in 2020 agreed to participate in this study. This was nearly half (55.1%) of the consults (n = 8,313) that were done in 2019 during the same period. The monthly variations in RT consultations for year 2020 and 2019 are shown in Figure 1A. Paradoxically, there was a 10% increase in consultations in March 2020 as compared with 2019. In May and June, we recorded the lowest number of consultations (approximately 70% lower than 2019) in alignment with the lower patient registrations and the initial phase of total lockdown with impaired transport. The numbers gradually increased from July after the restrictions were eased out in a phased manner from June. The reduction in patient numbers was not uniform across all disease sites. Figure 1B shows that as compared with 2019, in 2020, the proportion of patients receiving RT declined for all disease sites except breast (an increase from 17% to 24%) and pediatric, hematolymphoid, and bone soft tissue (an increase from 15.5% to 17.4%). Patients who cannot be accommodated at our center or who wish to receive RT closer to home are provided with a formal treatment referral letter. Such referrals also reduced drastically to 16.5% in 2020 compared with 44.3% in 2019 (Fig 1C).

### Study Population

Table 1 shows the demographic and treatment details of patients from 2020. The median age of the cohort was 52 years (interquartile range 41-61 years) with male and female sex nearly equally distributed. Nearly 81% of the patients who came for RT consultation preferred to take treatment at our center despite the pandemic and lockdown challenges. Nearly 50% of our patients hailed from outside the state of Maharashtra, whereas a minority came from foreign countries. For patients who originally intended to receive treatment closer to home but were stranded in Mumbai because of travel restrictions, their planned RT was started and completed without interruptions at our department.

**TABLE 1:**
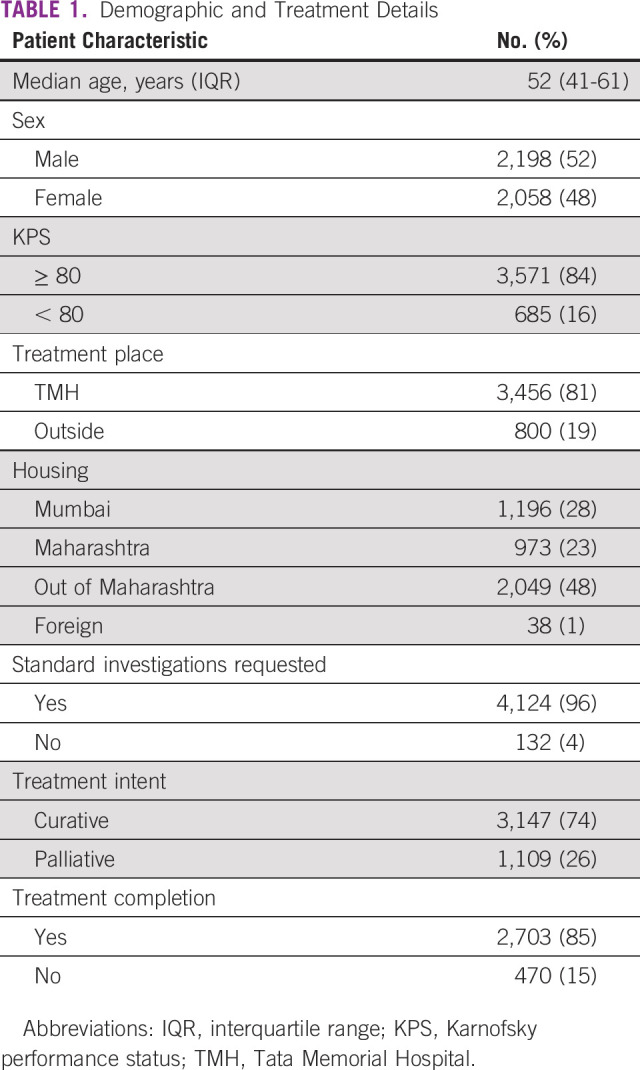
Demographic and Treatment Details

### Impact on Staging and Pretreatment Investigations

Standard investigations labeled essential as per the NCG management guidelines were followed for almost all the patients during the pandemic except for 4% of patients who did not undergo investigations such as endoscopic bronchial ultrasound and pulmonary function test for lung cancer management. Direct laryngoscopy using the Hopkins's rod lens for patients with head and neck cancer was performed judiciously using all precautions.

### Treatment Patterns

During the study period in 2020, approximately 74% of the patients were treated with curative intent and the rest with palliative intent. Among them, 85% could complete, whereas 15% patients were unable to complete their intended treatment. The main reasons for not completing the planned treatment were isolation because of COVID positivity, fear and anxiety of contracting the infection, inability to commute to the hospital, difficulty in bringing care givers, and lack of support staff such as medical social workers who help the financially weaker patients.

The change in RT practice was documented for all the disease sites. Concurrent chemotherapies were administered on the basis of the risk-benefit ratio. The most common change was the adoption of moderate to ultra-hypofractionated RT regimens in both curative and palliative settings.

Moderate hypofractionation has been adopted as standard for tumors with a low α/β value, such as breast and prostate cancers. Therefore, prepandemic, all patients with breast cancer received 40 Gy/15 fractions to the breast/chest wall with or without nodal targets, followed by a sequential tumor bed boost when indicated to a dose of 12.5 Gy/5 fractions. This changed during the pandemic as we adopted the five-fraction weekly or daily regimen for most of our patients on the basis of the international guidelines recommendations and the timely publications of the UK-FAST and FAST-Forward study.^15,16^ To further shorten the treatment duration, simultaneous integrated boost was used. This helped to reduce the number of hospital visits for RT from 20 to 5 for those with conserved breast cancer or from 15 to 5 postmastectomy. Thus, the largest reduction in the average number of fractions per patient was seen in patients with breast cancer, followed by patients with genitourinary cancer (Table 2). It is noteworthy that breast disease site had the least reduction in the number of patients (−11.5%) compared with other disease sites.

**TABLE 2:**
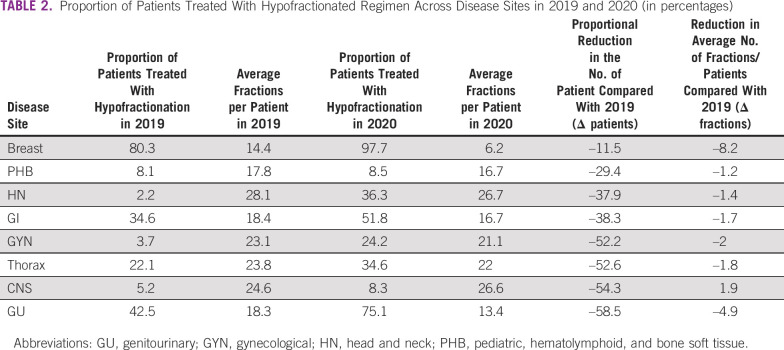
Proportion of Patients Treated With Hypofractionated Regimen Across Disease Sites in 2019 and 2020 (in percentages)

**FIG 2:**
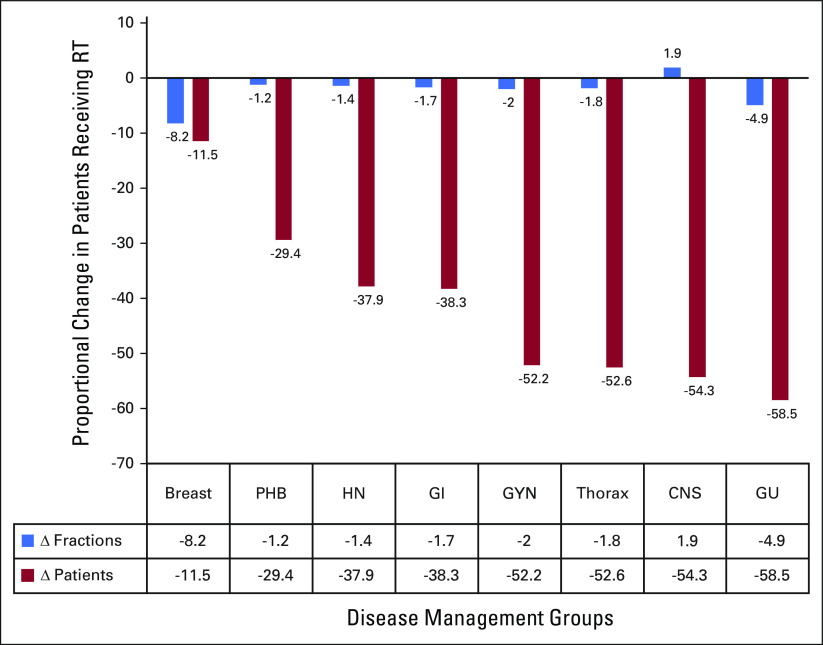
Site-wise reduction in the number of fractions versus the caseload in 2020 compared with the same period in 2019. GU, genitourinary; GYN, gynecological; HN, head and neck; PHB, pediatric, hematolymphoid, and bone soft tissue; RT, radiotherapy.

Most disease sites used modest hypofractionation as per the available evidence leading to an overall reduction in the average number of fractions per patient in 2020 compared with 2019 (Table 2). Figure 2 provides a visual comparison of the fall in the number of fractions against the fall in the percentage of patients. For most patients with gynecological cancer, brachytherapy delivery of multiple fractions was completed with a single intracavitary and interstitial implant.

### Impact of Testing COVID Positive

In total, 1,090 of 4,256 study patients (25.6%) underwent COVID testing before brachytherapy or if they had COVID-like symptoms. Of the 1,090, 146 patients (13.4%) tested positive for COVID while on RT. The highest number of COVID-positive patients belonged to the head and neck disease site (n = 45, 30.8). The disease site–wise proportion of the COVID-positive patients is given in Table 3.

**TABLE 3:**
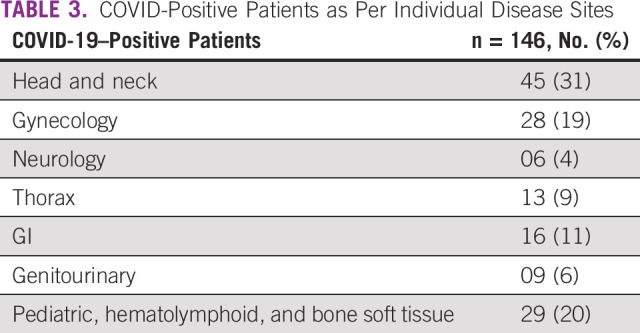
COVID-Positive Patients as Per Individual Disease Sites

Among those who tested COVID-positive, 30 (20.5%) could not complete their planned treatment. Table 4 shows that most of these patients did not complete their planned treatment because of reasons other than COVID-related morbidity or mortality. Almost 40% patients experienced disease progression while waiting for RT or during RT. Only 5 of 146 patients died due to COVID. Patients who tested COVID positive close to their RT completion and had a treatment gap of > 1 month were concluded early. In a small proportion of patients (4.8%), the reasons for RT discontinuation were not available as they were lost to follow-up. Of the total COVID-positive patients, only 60 patients (41.1%) are alive, 50 (34.2%) have died, and 36 (24.6%) are lost to follow-up.

**TABLE 4:**
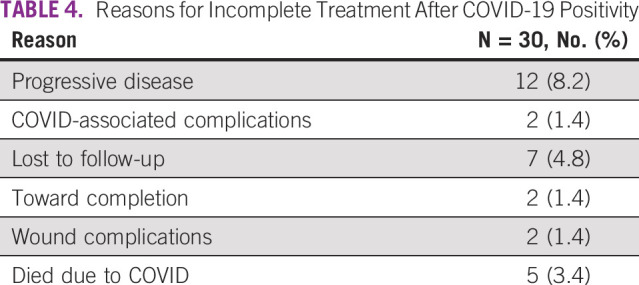
Reasons for Incomplete Treatment After COVID-19 Positivity

## DISCUSSION

Our study showcases the impact the early phase of COVID-19 pandemic had on the RT practice in India supported by data. Most studies published on similar topics have been opinion pieces or survey reports. In contrast, to our knowledge, ours is a unique study where we objectively recorded the pandemic-driven changes in the number, throughput, infection rates, and RT practice as compared with the same period in 2019. The dramatic reduction in the patient numbers (11,000 *v* 30,000) mostly during the early months of the lockdown shows the far-reaching impact of travel restrictions, disruption of care pathways, and the fear of infection in the absence of effective therapy or vaccines.

Our hospital provides highly subsidized cancer care, and, therefore, socioeconomically constrained patients from our state and across India (outside Maharashtra: 48%) prefer to receive treatment here. Most of these patients commute to and from the hospital using public transport. The strictly enforced ban on use of public transport to avoid crowding and spread of air-borne infection was the most common reason for preferring to receive treatment at centers closer to home during the lockdown.

Our center accommodated all patients who were stranded in Mumbai with little choice on where they would receive RT and even those who were receiving RT at centers who got abruptly converted to COVID specialty hospitals or were closed because of staff shortage. Staff rotations were made such that some of them were reserved as contingency by rotation. This was possible only by reducing the machine time from 13 hours to 10 hours. These rotations, travel restrictions, and mandatory quarantine or isolation for high-risk contact and COVID positivity of staff strained the system forcing others to compensate by working for 10-12-hour shifts. This was possible because of extraordinary understanding and commitment to treatment that our staff exhibited. None of the patients were sent back without treatment because of staff shortage.

Despite these challenging circumstances, of the 4,256 patients, nearly 85% completed the planned treatment. Nevertheless, this 15% noncompletion rate was nearly 6 times to that of our institutional standard of < 3%.^17^ The three main reasons for not completing the planned treatment were isolation because of COVID positivity, fear and anxiety of contracting the infection, and inability to commute to the hospital. This again shows the importance of social, psychological, and financial support that is required to complete planned treatments. Pretreatment and staging investigations were not compromised, except for aerosol generating procedures in accordance with several published guidelines.^18^

Various expert groups published practice modifications during the pandemic aimed at providing therapies with survival benefit especially to those who would have been most affected by treatment delays.^13,19-22^ This may have been an important reason for the drop in the genitourinary caseload (-58.5%) where RT was deferred by using hormonal therapy. Modest hypofractionation was used during the pandemic in many disease sites including head and neck, gynecological, thorax, and rectal cancers and was instrumental in reducing the hospital visits and keeping the infection rate low (13.4%).^23-27^ Patients with breast cancer comprise 17%-20% of our caseload. The universal use of ultra-hypofractionation for adjuvant breast RT helped to accommodate most of the patients while allowing implementation of COVID-specific sanitation and social distancing protocols.^12,15^ Since breast is a surface organ, surgery was not contraindicated. This led to comparatively lower reduction in the adjuvant therapy caseload among patients with breast cancer compared with the other sites. Using five-fraction regimen was especially beneficial to accommodate these patients. Single-fraction or short-course palliative treatments were recommended by various international guidelines and were widely adopted to reduce the hospital visits. Changes in the brachytherapy protocols helped to reduce the burden of COVID testing and hospital admission especially when there was a severe shortage because of wards being converted into isolation facilities. This also helped to minimize the overall treatment time.

Despite these measures, 13% patients tested positive. About one third of the COVID-positive patients were patients with head and neck cancer who may have undergone a more frequent COVID testing possibly because of similar symptoms they share with COVID. However, the high proportion of positivity could be from the difficulty in achieving appropriate mask fit, especially with advanced disease and nasogastric tubes in situ. Mandatory COVID testing before brachytherapy identified nearly 19% of asymptomatic patients with gynecological cancer infected with SARS CoV-2 infection leading to increased overall treatment time. The impact of this delay on outcomes is being studied in another prospective cohort study. Although single application of multiple treatments may have helped patients and the hospital, RO resident's hands-on brachytherapy training was jeopardized because of a decrease in patient load and intracavitary or interstitial procedures (two-three procedures *v* six-eight procedures/day before pandemic) and will have a far-reaching impact. Similarly, clinical research was also affected because of the lower accrual of patients in RT clinical trials. It is noteworthy that none of the patients with breast cancer were diagnosed with SARS CoV-2 infection during RT. This may largely be attributed to the use of ultra-hypofractionated RT and minimized hospital visits.

Our hospital policy during the study period mandated two successive negative COVID RT-PCR reports for all patients and staff before they could resume their treatment or duty at our hospital. This may have been among the important reasons for 8% of our COVID-positive patients showing disease progression after unplanned and prolonged treatment interruption. Only a small proportion of study patients (1.4%) experienced COVID-related complications in the form of pneumonia and lung fibrosis, and 3.4% ultimately died due to the disease showing that RT was indeed safe to deliver to the majority of patients. Approximately 80% of the patients completed planned treatment after testing negative for infection. These results are noteworthy for pandemic preparedness in future.

Our study was not designed to capture the details of conspicuously aggravated financial and social constraints faced by our patients. The pandemic restricted the funds that were previously received by our department to support poor patient's treatment through the corporate social responsibility scheme further burdening the patient and their caregivers. Adding to these challenges were the financial constraints faced by the hospital in implementing additional sanitization, free COVID testing, and staffing protocols against the sharp fall in revenues because of the fewer patients undergoing treatment at our hospital.

The pandemic had far-reaching implications in multiple domains of life, viz, social, economic, emotional, and mental health not only of patients and caregivers but also of health workers. A multinational survey of our hospital to study the mental impact on health care workers revealed significant levels of anxiety, depression, and stress among the RO fraternity and yet there were no treatment interruptions because of staff unavailability.^28^

The strength of our study is in the number of patients evaluated across all disease sites showcasing, to our knowledge, the largest departmental data on the deleterious impact of the pandemic reported till date. Another strength lies in providing a comparator cohort of 2019 which allows to objectively assess site-wise and overall changes in patient number and RT practice. Although simple in design, it had some limitations such as the retrospective nature of this study, lack of toxicity data from hypofractionated treatments, survival outcomes, direct and indirect cost analysis, and paucity of data on treatment patterns of patients who were referred to other centers.

Against continuing adversities, our department still continues to honor our hospital's motto of service, education, and research. The lessons learned during the first wave of the pandemic helped us prepare better for the second wave. With the ongoing steady vaccination strategies and following all COVID protocols, we expect that patients will be able to attend clinics and complete their treatment as planned.

In conclusion, during the first wave of COVID-19 pandemic in India, despite grave challenges, TMC, Mumbai, continued to offer radiotherapy. Hypofractionation was encouraged and adopted across various disease sites to minimize multiple visits. COVID positivity led to a delay in completing radiotherapy, whereas a minority were unable to complete the planned treatment because of prolonged COVID-19 infection and government-imposed restrictions consequent to the lockdown across the country. Future studies should be aimed at evaluating outcomes consequent to implemented pandemic-specific modifications in RT practice.
